# Video-assisted thoracoscopic cardiac denervation of refractory ventricular arrhythmias and electrical storms: *a single-center series*

**DOI:** 10.1186/s13019-019-0838-6

**Published:** 2019-01-21

**Authors:** Luis Jaime Téllez, Juan Carlos Garzón, Eric Edward Vinck, Julian David Castellanos

**Affiliations:** 1 Department of Thoracic Surgery, Fundación Cardioinfantil, Bogotá, Colombia; 2 Department of Surgery, El Bosque University, Av. Cra 9 No. 131 A - 02, Bogotá, Colombia; 3 Department of Surgery, Universidad Nacional, Bogotá, Colombia

**Keywords:** Ventricular arrhythmias, Electrical storms, Denervation, Video-assisted thoracic surgery, Chagas, Implanted cardioverter defibrillator, Sympathectomy

## Abstract

**Background:**

The combined treatment of beta-blockers with ablation and Implanted cardioverter defibrillation therapy, continues to be the mainstay treatment for ventricular arrhythmias (VAs). Despite treatment, some patients remain refractory.  Recent studies have shown success rates using video-assisted thoracoscopic (VATS) cardiac denervation as an effective therapeutic option for these patients.

**Case series presentation:**

During a period of three years, from 2015 through 2017, twenty patients (*N* = 20) failed traditional medical and interventional treatment for the management of ventricular arrhythmias and electrical storms. After remaining refractory, the patients were referred to our thoracic surgery department for a VATS based treatment. The patients all had ventricular arrhythmias and electrical storms secondary to different cardiomyopathies. The patients were refractory to combined medical (beta-blockers), Implanted Cardioverter defibrillation (ICD) and ablation therapy. All twenty patients agreed to surgery and were taken to cardiac denervation using a bilateral VATS approach by two thoracic surgeons at a single Cardiothoracic center. During the month prior to bilateral VATS denervation a combined total of twenty-nine (*N* = 29) ICD shocks were registered in addition to six (*N* = 6) cases of electrical storms averaging three (*N* = 3) shocks per day. Mean shocks per patient was 2.3. During the first three months following VATS, the patients had a 90% (*N* = 18/20) total resolution of ICD registered shocks, a 100% (N = 6/6) resolution of electrical storms, and a 92% (*N* = 11/12) resolution of shocks in patients having previous ablation therapy. No complications were documented following surgery except for one case of pneumothorax as a result of the procedure, and there were no peri-operative mortalities.

**Conclusions:**

Bilateral thoracoscopic cardiac denervation can be a safe and seemingly effective therapeutic option for patients presenting with life-threatening refractory ventricular arrhythmias and electrical storms in a variety of cardiomyopathies including Chagas disease.

## Background

Thoracoscopic sympathectomy was first described in 1942, and the first clinical reports date back to 1961 by Estes and Izlar. Left cardiac denervation was first reported in 1971 by Moss and McDonald and the first case of video-assisted thoracoscopic left sympathectomy was reported by Reardon and colleagues in 2000 [1–4]. Three years later Li and colleagues presented the first small case series [2–4]. During the last 5 years, VATS cardiac denervation has become an accepted treatment option for refractory ventricular arrhythmias secondary to ischemia, hereditary arrhythmias, channelopathies, and arrhythmias due to long-QT-syndrome (LQTS) [2–6]. Most studies show a 70–80% effectiveness within the first month to 1 year following VATS cardiac denervation [1–6]. Although the majority of cases are left ventricular arrhythmias, right-sided VAs may also benefit from this treatment option.

The sympathetic nervous system regulates cardiac electrophysiology through interconnections between preganglionic axons in the sympathetic paravertebral chains. The preganglionic axons synapse on neurons within the stellate ganglion (fusion of the lower cervical C7 and T1 ganglia) and run through T2–T4 [2–8]. These interconnections are an important component in the generation of life-threatening ventricular arrhythmias. Because of this fusion at the stellate ganglion, it is believed that cardiac impulses and arrhythmias may be generated even from C7; effective antiarrhythmic VATS therefore requires proper dissection of the lower half of the stellate ganglion in order to achieve the desired results with cardiac denervation [3–9]. The treatment of ventricular arrhythmias aims to prevent degeneration of cardiac myocytes, prevent development of heart failure and sudden cardiac death. Catheter ablation therapy is indicated for patients presenting with uncontrolled non-sustained VTs, and sudden cardiac death may be prevented using implantable cardioverter defibrillators. Inappropriate shocks however, lead to cardiac myocyte damage in turn increasing the risk of sudden cardiac death [2–7]. Patients who are unresponsive to combined medical (beta-blockers), ablation and ICD therapy and those presenting with electrical storms are considered candidates for a VATS based therapy [4–10]. This manuscript describes a series of twenty patients having had bilateral VATS cardiac denervation for refractory ventricular arrhythmias.

## Case series presentation

During a period of three years, from 2015 through 2017, twenty patients (*N* = 20) failed traditional medical and interventional treatment for the management of ventricular arrhythmias and electrical storms. After remaining refractory, the patients were referred to our thoracic surgery department for a VATS based treatment. The patients all had ventricular arrhythmias and electrical storms secondary to different cardiomyopathies. All were refractory to combined medical-beta-blocker therapy (primarily amiodarone), implanted cardioverter defibrillation (ICD) and ablation therapy. The inclusion criteria for considering VATS denervation in these patients included ventricular arrhythmias refractory to combined pharmacological (beta-blocker), ablation and ICD therapy, and patients presenting with electrical storms defined as three or more registered ICD shocks within a 24-h period. All twenty (*N* = 20) patients agreed to surgery and were taken to cardiac denervation using a bilateral VATS approach by two thoracic surgeons at a single Cardiothoracic center. Of these 20 patients (14 men and 6 women) ages 20 to 73 years (average 56.7 years), nine (*N* = 9) had VAs secondary to Chagas disease, seven (*N* = 7) patients secondary to chronic ischemic cardiomyopathy, three (*N* = 3) patients had VAs secondary to dilated cardiomyopathy and one (*N* = 1) patient had VAs secondary to long-QT syndrome. Fifteen (*N* = 15) patients registered sustained ventricular tachycardias (VT), three (*N* = 3) patients registered ventricular fibrillations (VF), two (*N* = 2) patients registered mixed VT/VFs and six (*N* = 6) patients had electrical storms. All twenty (*N* = 20) patients had beta-blocker (amiodarone) therapy, six (*N* = 6) had pacemakers and nineteen (*N* = 19) had Implanted Cardioverter defibrillators (ICDs). Of the twelve (*N* = 12) patients having had previous ablation therapy, 92% (*N* = 11) had ICDs. Patient comorbidities included arterial hypertension primarily and six (*N* = 6) were on the heart transplant waiting list. A summary of patient characteristics is detailed in Table 1. The patients presenting with VAs secondary to Chagas disease all had their Trypanosoma cruzi treated with anti-protozoan therapy, however their resulting cardiomyopathy progressed to refractory VAs.

**Table 1:**
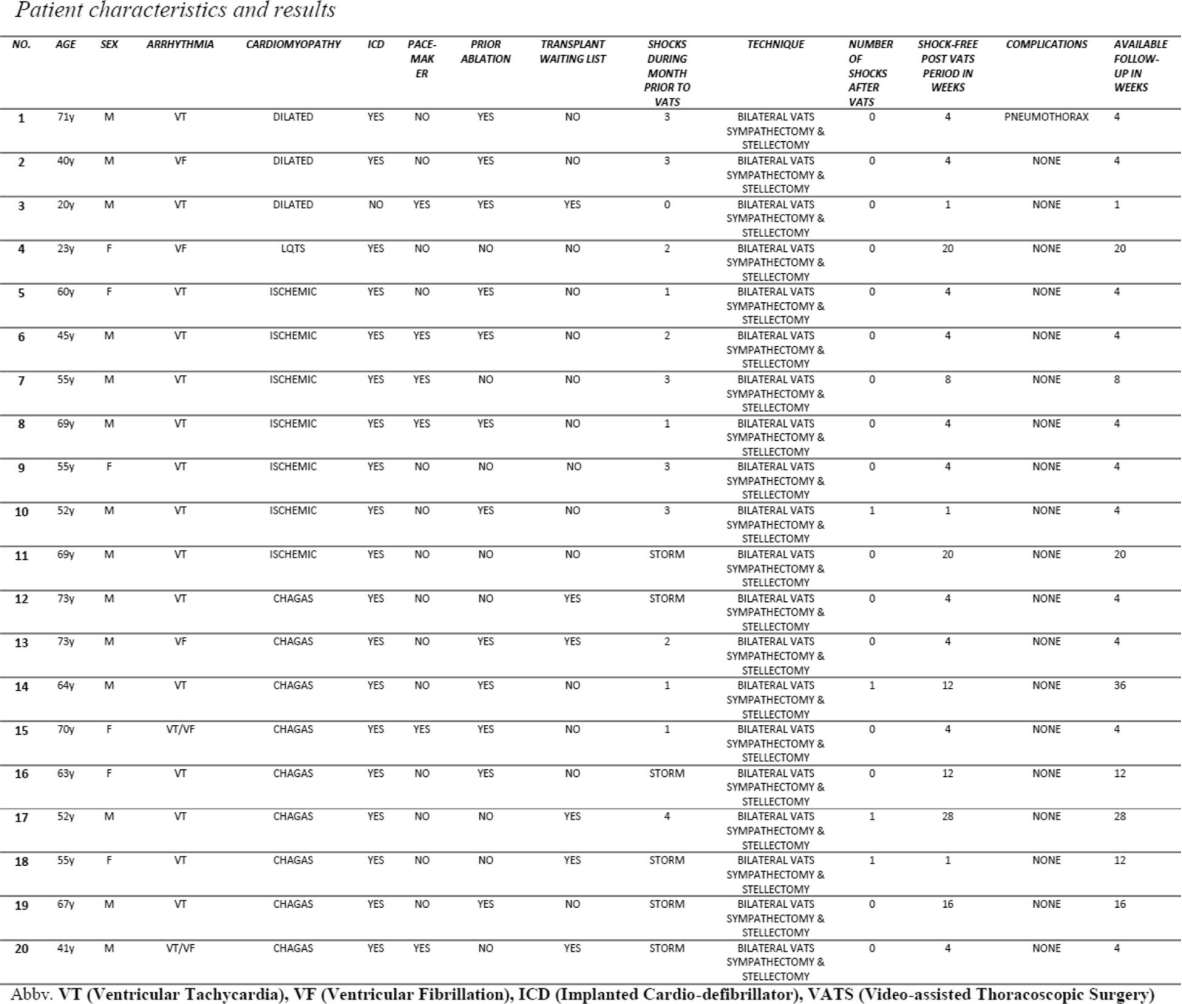
Patient Characteristics and Results

## Surgical technique

**Fig. 1:**
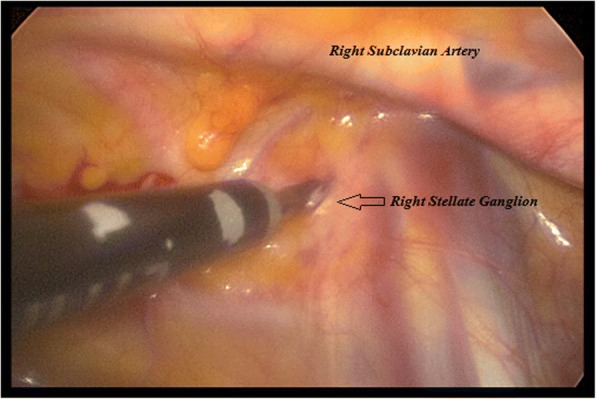
Right Sympathetic chain dissection at the Stellate ganglion (sympathectomy & inferior hemi-stellectomy)

**Fig. 2:**
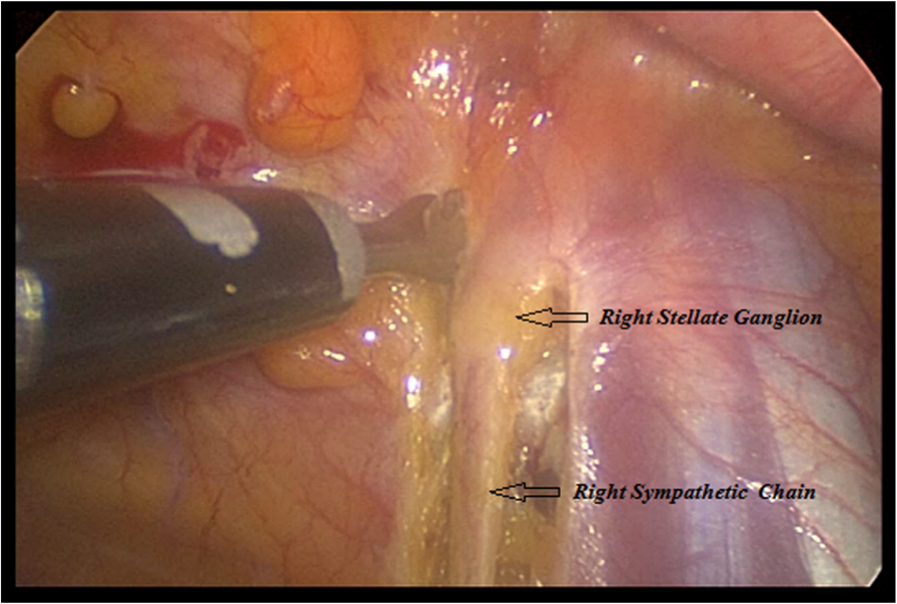
VATS Sympathetic chain dissection (sympathectomy & inferior hemi-stellectomy)

**Fig. 3:**
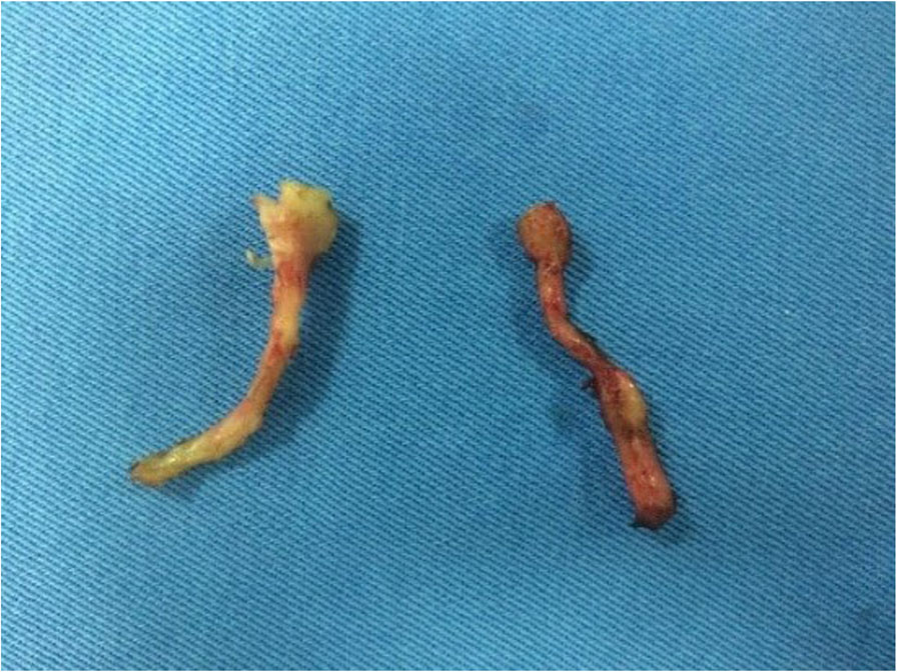
Resected T1-T4 bilateral sympathetic chains with lower half of the Stellate ganglia

A bilateral VATS approach was performed in all twenty (*N* = 20) cases. The patients were placed in a 45-degree supine position with both arms in upper extension. Using combined inhaled and IV general anesthesia, along with single lung intubation, a bilateral two-port VATS approach was performed. The surgical technique involved active lung collapse using intrapleural CO2 of up to 8 mmHg, and a 3rd and 4th intercostal space with an anterior and medial axillary line technique. All procedures were started on the left side to guarantee a left sided treatment in case the patients did not tolerate the entire bilateral surgery. A Harmonic cutter was used to dissect the lower half of the stellate ganglion (T1) down to T4 (inferior hemi-stellectomy and sympathectomy). Figures 1 and 2. Considering possible anatomical variations such as the Kuntz nerve, a transverse dissection below the second intercostal space was performed [10, 11]. Fig. 3. Average surgery time was 38 min for the twenty patients and no chest tubes were routinely placed at the end of the procedures. All resected sympathetic chain specimens were sent to pathology and the neuronal tissues were confirmed. Follow-up was conducted by the Electrophysiology & Arrhythmia department registering post-op ICD shocks.

## Results

During the month prior to VATS denervation a combined total of twenty-nine (*N* = 29) ICD shocks were registered in addition to six (*N* = 6) cases of electrical storms which averaged three (*N* = 3) shocks daily. Total mean shocks per patient was 2.3 for an approximated 47 total registered shocks prior to surgery. During the first three months following VATS, the patients had a 90% (*N* = 18/20) total resolution of ICD registered shocks, a 100% (*N* = 6/6) resolution of electrical storms, and a 92% (11/12) resolution of ICD registered shocks in patients with previous ablation therapy. The follow-up period varied from one to nine months, during which a total of four (*N* = 4) ICD registered shocks were documented showing complete resolution in 80% (*N* = 16) of patients during this time frame. Two patients each had one (*N* = 1) shock within the week post-op, one patient had a single (*N* = 1) shock at three months post-op, and another patient had one (N = 1) shock at seven months post-op. During the first three months post VATS, only two (*N* = 2) registered ICD shocks were reported in all patients showing an overall 90% (*N* = 18) success rate along with complete resolution of electrical storms 100% (*N* = 6/6). A total number of pre-VATS shocks of (*N* = 47) including six (*N* = 6) cases of electrical storms were reduced to only two (*N* = 2) shocks during the first 3 months following VATS and complete resolution of electrical storms during the entire follow-up period. All four (*N* = 4) registered ICD shocks after VATS denervation in all twenty patients were VTs, no new VFs or electrical storms were registered. Results are summarized in Table 1.

The twelve (*N* = 12) patients remaining refractory following ablation therapy had a 92% (*N* = 11/12) resolution of ICD registered shocks during the first three months following VATS. Of the nine (*N* = 9) patients with refractory VAs due to Chagas disease, five (*N* = 5) had electrical storms and the other four (*N* = 4) patients had a total of eight (*N* = 8) registered ICD shocks during the month prior to VATS. Only one (*N* = 1) shock was registered during the first three months following VATS for an 89% (N = 8/9) resolution and complete resolution of electrical storms 100% (*N* = 5/5). Seven (*N* = 7) patients had refractory VAs secondary to Ischemic cardiomyopathy, of which one (*N* = 1) patient presented with electrical storms and the other six (*N* = 6) had a total of thirteen (*N* = 13) registered ICD shocks during the month prior to VATS. During follow-up only one (*N* = 1) shock was registered for a success rate of 86% (*N* = 6/7) as well as complete resolution of electrical storms 100% (*N* = 1/1). The three (*N* = 3) patients with refractory VAs secondary to dilated cardiomyopathy had a total of six (N = 6) registered shocks during the month prior to intervention. During the month following VATS, there was complete resolution of registered ICD shocks, 100% (*N* = 3/3) success. The one patient with LQTS had two (*N* = 2) registered shocks prior to VATS and none during follow-up, 100% (*N* = 1/1) resolution.

Complications using VATS denervation include Horner syndrome, nocturnal anisocoria, and compensatory hyperhidrosis. These complications can be prevented by avoiding the use of electrocautery, and properly dividing the lower half of the stellate ganglion [11–14]. None of these were reported in our patients, and no significant neuropathic pain was observed during the post-op period. One patient needed a chest tube due to pneumothorax secondary to the procedure and one patient died at 3 months post-op due to the natural course of Chagas disease. One male patient did not have an ICD and post-op follow-up was conducted through symptom referral.

## Discussion

Immediate response to VATS cardiac denervation can be evaluated comparing pre-surgery to post-operative ICD shocks. Recent reports and series show positive results using VATS cardiac denervation in both bilateral as well as unilateral (left sided only) approaches. Studies show a drop of 93% in ICD shocks and a reduction of electrical storms from 38 to 14% [8–13]. Some authors like Schwartz prefer a left-sided only approach arguing the dominance of the left sympathetic innervation. The left ventricle contributes to a significant catecholaminergic stimulus in the generation of refractory arrhythmias especially in long-QT syndrome. Authors like Ajijola consider a left-sided only approach ineffective in 50% of cases which later require an additional right-sided sympathectomy to effectively control VAs, predominantly in structural cardiomyopathies [12–14]. Richardson and colleagues reported a series of 7 patients with refractory VAs (median of 1 episode of sustained VA per-month) having no VAs during 7 months following VATS denervation [12–15].

In this series the number of ICD registered shocks during the month prior to VATS was compared to those during the month following cardiac denervation. During the first three months following VATS cardiac denervation, the patients had a 90% (18/20) total resolution of ICD registered shocks, a 100% (6/6) resolution of electrical storms, and a 92% (11/12) resolution of ICD registered shocks in patients with previous ablation therapy. This study showed an overall immediate short-term post-op success rate of 90% (18/20) during the first three months following VATS. The patient groups presenting the best response to VATS denervation were those with electrical storms 100% (*N* = 6/6) resolution, and those with prior ablation therapy 92% (*N* = 11/12) resolution. Although most of these patients did not have long-term follow-up, this study shows considerable results in the immediate post-op and short-term periods during the months following cardiac denervation in those whom follow-up was possible.

Patients suffering from refractory ventricular arrhythmias and electrical storms are high risk patients with exhausted treatment options. VATS cardiac denervation offers both a safe and effective last resort option for these patients with positive short-term outcomes. VATS sympathectomy, a thoracic surgical solution to a medical cardiological problem, may benefit these high-risk patients extending their therapeutic options. Taking into consideration the comorbidities and high sudden cardiac death risk in these patients, long-term follow-up is not always possible limiting the number of cases available in the literature. Additional research is warranted to assess long-term results. Small sample size and short follow-up periods were the primary limitations of this study.

## Conclusions

Bilateral thoracoscopic cardiac denervation can be a safe and effective therapeutic option for patients suffering from life-threatening refractory ventricular arrhythmias and Electrical Storms secondary to different cardiomyopathies including Chagas disease. However, because of variable results in the literature, further controlled studies and long-term follow-up is necessary to determine optimal technique effectiveness.

## Abbreviations

ICD: Implanted Cardioverter defibrillator
VA: Ventricular Arrhythmias
VATS: Video-assisted Thoracoscopic surgery
VF: Ventricular Fibrillation
VT: Ventricular Tachycardia
